# Does the construction of an integrated transport network promote urban innovation? A perspective based on the theory of flow space

**DOI:** 10.1371/journal.pone.0259974

**Published:** 2021-11-15

**Authors:** Shengrui Zou, Mingxian Li, Junfei Chen, Yixin Chen

**Affiliations:** 1 Business School, Hohai University, Nanjing, Jiangsu, China; 2 Yangtze Institute for Conservation and Development, Hohai University, Nanjing, Jiangsu, China; 3 Jiangsu Research Base of Yangtze Institute for Conservation and High-Quality Development, Nanjing, Jiangsu, China; Institute for Advanced Sustainability Studies, GERMANY

## Abstract

Transportation infrastructure, which has always been regarded as an important element to promote regional innovation, accelerates factor flows and productivity spillovers. In February 2021, the State Council of China issued the outline of national integrated multidimensional transportation network planning (2021–2050), which proposed that during the 14th Five-Year Plan period, the Yangtze River Delta would speed up the construction of an integrated transport network to serve the dual circulation development pattern in China. However, few studies have systematically investigated the development of integrated transport in the Yangtze River Delta, especially the relationship between transport operating efficiency and regional innovation based on the theory of flow space. This study aims to calculate the integrated transport efficiency of 26 cities in the Yangtze River Delta and analyse the spillover effect of efficiency improvement on urban innovation. The results reveal that integrated transport efficiency is relatively stable at approximately 0.92. We find that the local innovation value would increase by 0.119% with every 1% increase in transport efficiency, and it would exceed 0.26% after introducing spatial factors. The spillover effect on the surrounding cities is significantly higher than that in the cities themselves, and the result is 0.292 under the economic spatial distance weight matrix. These findings will support the construction of the integrated transport network and provide useful references for government decision makers in the Yangtze River Delta.

## Introduction

As a representative of the new economic geography, Castells opened a new era of studying urban innovation from the perspective of flow space by stating that material flows can realize space-time sharing without geographical proximity [1]. The theory of flow space mainly includes four aspects: network flow, network city, dual city and infinite time. Among these aspects, network flow explains the basic elements, such as talent, capital, information and other elements. Network city refers to a mobile network composed of cities, megalopolises or even countries in geographical space [2]. Dual city describes the dual attributes, that is, the economic attributes and social attributes of urban space. Infinite time studies the characteristics of the spatiotemporal compression effect [3]. The development of flow space provides a theoretical basis for the discussion of the spatial structure of innovation networks [4]. Scholars began to analyse the innovation network structure with cities or enterprises, universities, and public research and development (R&D) institutions as nodes [5]. Compared with other types of nodes, cities, as the intersections of various flows, can better avoid knowledge redundancy and obtain heterogeneous external knowledge [6–8], thus becoming the most concerning research object.

When scholars attempt to adopt flow data to evaluate the structure of urban innovation networks, traffic data become an important data source because of their importance and convenience [9, 10]. To the best of our knowledge, the construction of transportation infrastructure can reduce transportation costs and improve spatial accessibility, leading to the free flow of innovation elements and cross-regional innovation spillover. The initial studies estimated traffic data using a logical reasoning model [11] while later methods mainly include actual measurement and parameter substitution [12]. The relevant studies generally focused on a specific form of transportation, such as urban buses, suburban railways, urban ports, and road freight, to discuss the functional relationship between cities [13]. Among the research on the various forms, the research on high-speed railways is particularly abundant. Albalate and Tomer proposed that high-speed railways can accelerate accessibility between cities, bring about the time-space compression effect, and reshape the factor spatial structure and economic distribution [14]. By studying the spatial expansion of high-speed rail networks in China, You et al. found that the connectivity between Chinese cities has been strengthened and the hierarchical structure of urban networks has been gradually homogenized [15].

The reform of information technology promotes the rapid expansion of transportation infrastructure. The rapid development of integrated transport in foreign countries in recent years has brought new opportunities for economic growth to many cities, such as Chicago, Kyushu, Berlin and others [16]. Barnum et al. proposed that only when considering the technical and resource allocation status of each major transportation type can the urban innovation of metropolises be estimated effectively [17]. Therefore, the concept of integrated transport has been widely studied because integrated transport can make full use of the advantages of various transportation modes using optimization schemes. The integrated transport industry conforms to the networking trend according to the theory of flow space by covering all forms of transportation related to cities, including the four subsystems of railways, highways, harbours and airports [18].

Although the relevant research in China lags behind that in developed countries, with the continuous increase in the demand for convenient transportation, China is constructing an integrated transport system by constantly referring to the experiences of foreign countries. The 13th Five-Year Plan for the development of a modern integrated transport system issued by the State Council of China in 2017 clearly proposed that China would promote the further integration of different means of transport and build a safe, convenient, efficient, green, and economical modern transport system [19]. In February 2021, the outline of the national integrated multidimensional transportation network plan (2021–2050) further proposed strengthening interconnections and network resilience to serve the dual circulation development pattern in China [20]. Generally, if the transportation network leads to a one-way flow of innovation elements, it has a siphon effect on urban innovation, that is, a negative spatial spillover effect. If the transportation network results in the two-way flow of innovation elements, it represents a diffusion effect of innovation, which results in a positive spatial spillover performance. Some positive effects in this field have been gradually emerging. Matsumoto et al. used traffic data including port throughput, highway traffic volume and railway traffic volume to study the interaction between cities and revealed how globalization affects the status of cities in different development stages, thus changing the regional spatial pattern [21]. Donaldson and Hornbeck concluded that integrated transport shortens the space-time distance between cities [22], which is one of the key factors that influences the innovation of urban agglomerations in China [23]. With the continuous construction of integrated transport, the hidden barriers of the geography, culture and markets among cities in China will be gradually eliminated, showing an obvious trend of integration and balanced development [24].

Through the analysis above, we believe that with the continuous enrichment of the integrated transport industry, urban agglomeration closely integrates various resources into a larger scope, and the spatial correlation effect of innovation elements is becoming increasingly significant. Although there are many studies on the network structure of regions, studies on the social and economic impacts of the network structure, especially regarding the spatial features of regional innovation, are relatively limited. Moreover, most studies focus on a specific transportation mode when studying the impact of transportation on urban innovation. The construction of an integrated transport system plays a multilevel and more critical role in promoting regional innovation. Therefore, based on the theoretical analysis of flow space, this paper studies the impact of integrated transport systems composed of multiple transportation modes on urban innovation and considers spatial factors in the model. Since there are few relevant studies on the Yangtze River Delta, this research benefits the Yangtze River Delta by providing policy suggestions to coordinate regional innovation and implement innovation-driven strategies.

The remainder of the paper is organized as follows. The next section briefly discusses the conceptual framework. The third section introduces the study area, constructs the model, and discusses the selection of the variables and data sources. The fourth section estimates and analyses the integrated transport efficiency of 26 cities in the Yangtze River Delta and explores its spatial spillover effect on regional innovation using the spatial econometric model. The fifth part discusses the changes and influence of the scope in the Yangtze River Delta. Finally, this paper summarizes the main findings and provides some suggestions for decision-making departments.

## Conceptual framework

According to the theory of flow space, the essence of urban innovation is the process of innovation value flowing and transferring between different nodes. There is a typical technology gap within and between cities because of the influence of the economic base, cognitive distance and the diffusion channel [25]. Knowledge flow, which leads to urban innovation, relies on face-to-face communication [26, 27], is assigned and changed by different nodes in the process of transmission, and finally materializes into products [28, 29].

Within a city, the construction of an integrated transportation network can improve transportation efficiency and reduce production costs so as to improve the accessibility of productive inputs and intermediate products. In addition, considering that the spillover of knowledge and technology has time and space limitations, the potential advantages of enterprises depend on the degree of asymmetry in the technological capability of the cluster [30]. Geographical proximity helps enterprises improve the efficiency of knowledge exchange through market mechanisms [31]. Heterogeneous skilled workers form technological clusters in the interaction process, accompanied by the diffusion of knowledge [32]. Therefore, regarding the geographical exclusiveness or high stickiness of knowledge, an integrated transport network can promote the formation of a local agglomeration effect, thus changing the spatial distribution of industries [33].

**Fig 1:**
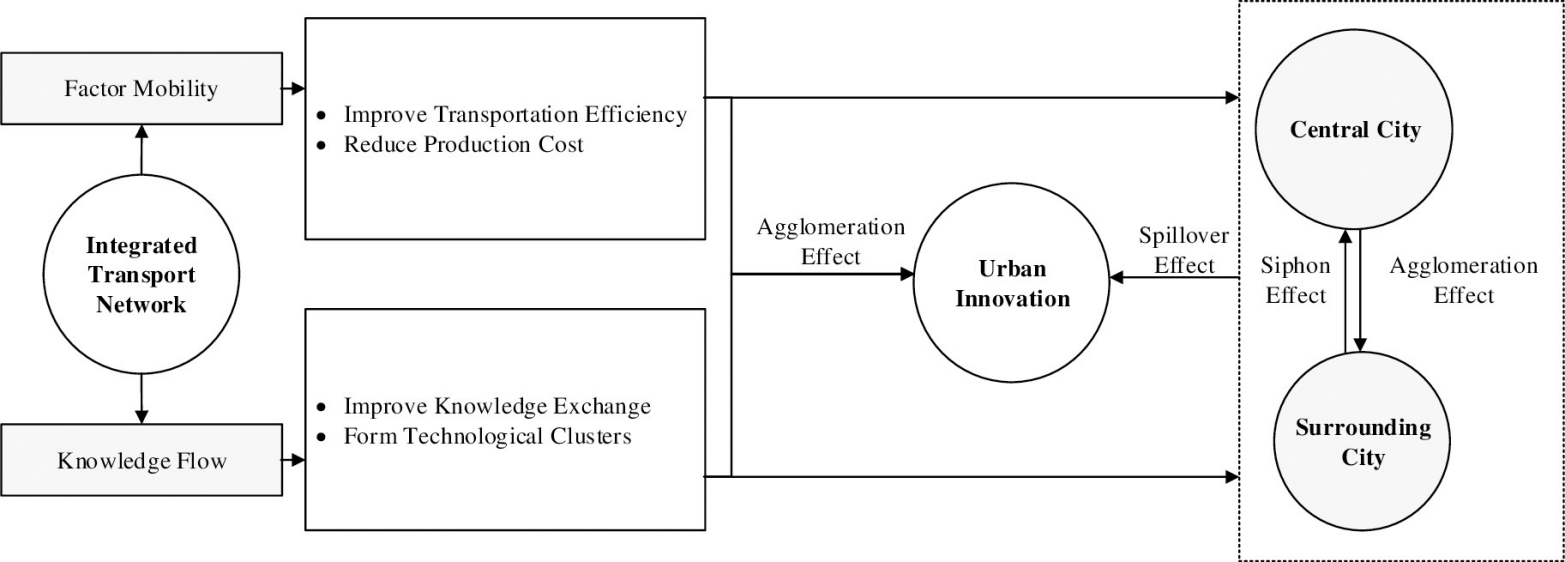
Conceptual framework of integrated transport network influencing urban innovation.

Between cities, the spatial choice of innovation elements depends on the spatial structure and R&D environment, especially the relative local profits and rent costs. Specifically, some high-tech elements usually choose central cities with higher pay and higher rent costs [34]. Because innovation elements always flow to regions with advanced technology [35], the development of integrated transport systems improves the mobility of elements and reduces the flow costs of products. With the sharp decline in space-time costs and the expansion of the labour market, high-tech elements in central cities obtain greater returns by expanding their production modes [36]. If the innovative elements only move to a central city in one direction, a siphon effect of the central city will appear. However, the relatively low-tech elements in the surrounding areas can improve allocation efficiency through complementary factor supply, which may form economies of scale; therefore, the agglomeration effect is expected to be greater than the siphon effect, thus providing a positive incentive for innovation activities [37]. As time passes, traffic and market barriers between regions will be gradually eliminated, the market will present an obvious trend of integration, and the spatial spillover effect will be enhanced. To conclude, the integrated transport network can accelerate the orderly flow of innovation elements, increase face-to-face communication, change the intensity of knowledge connections between regions, and ultimately promote innovation in local and adjacent regions [38]. The specific conceptual framework is shown in Fig 1.

## Materials and methods

### Study area

The economic growth rate in the eastern coastal areas of China is higher than that in the inland areas. With the release of a series of policy documents, the eastern areas have taken the lead in developing an integrated transport network in the area. After more than ten years of rapid development, the total number of transportation facilities in the eastern region has increased significantly, and the efficiency of the integrated transport system has improved significantly, especially in the Yangtze River Delta.

**Fig 2:**
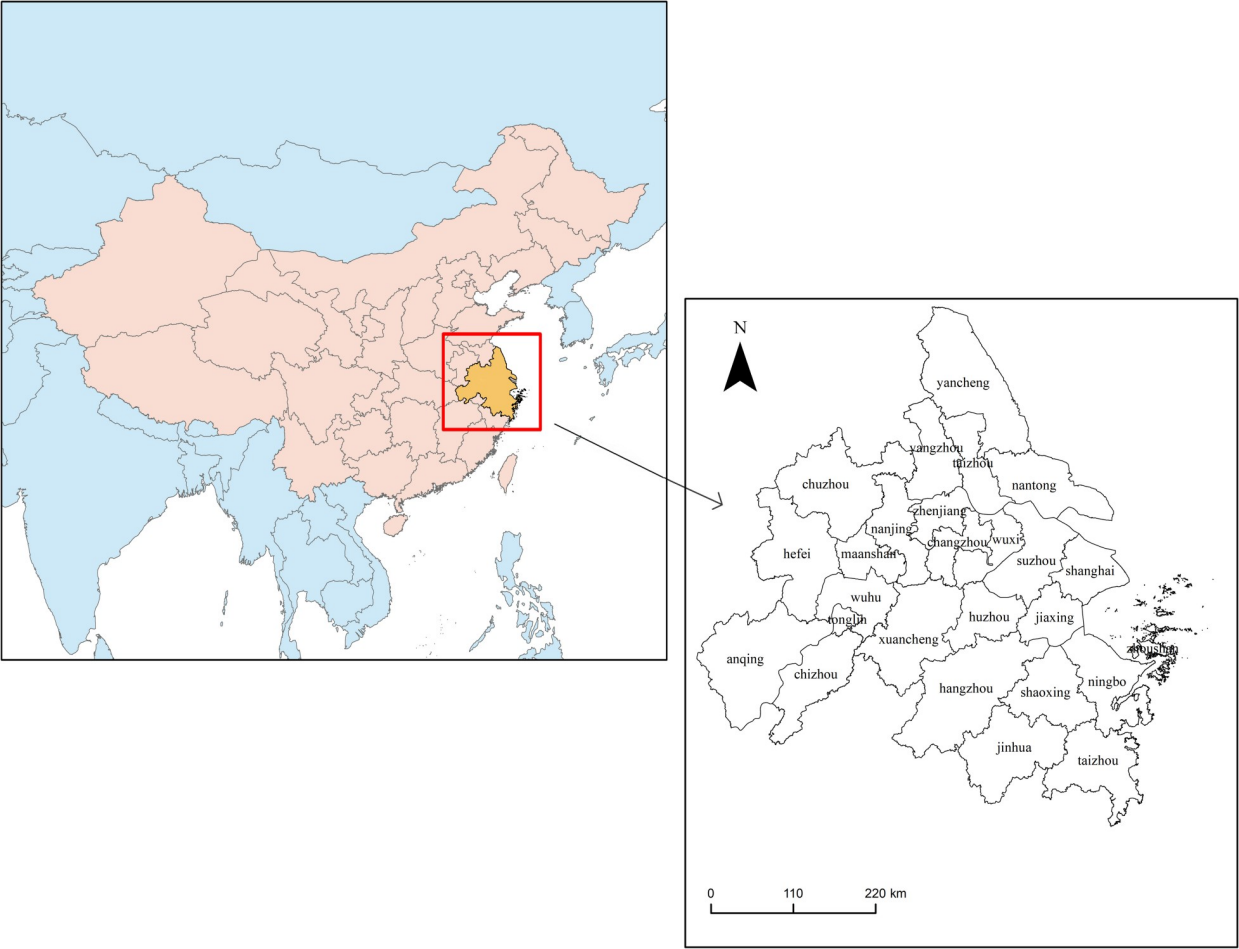
Location of 26 cities in the Yangtze River Delta. (The left frame is a part of the world map, in which the red and yellow parts belong to China, and the yellow part is the study area. The right frame is to enlarge the yellow area of 26 cities in the Yangtze River Delta. The data come from publicly available data on the internet and are organized by the authors themselves. The figure is drawn by Arc GIS).

The Yangtze River Delta is one of the most active, open and innovative regions in China and plays a significant strategic role in the overall situation of national modernization and all-round opening-up. The Yangtze River Delta is located in the plain area downstream of the Yangtze River and has favourable conditions for opening up along the coast and the river. The Yangtze River Delta is composed of many cities within the scope of Shanghai, Jiangsu Province, Zhejiang Province and Anhui Province, with Shanghai as the centre. The integration of the Yangtze River Delta began in 1982, and the process has continued for more than 30 years. The spatial scope of Yangtze River Delta urban agglomeration has been changing and adjusting. According to the development plan of urban agglomeration in the Yangtze River Delta (2016) issued by the National Development and Reform Commission in China, there are a total of 26 cities in the Yangtze River Delta [39], including Shanghai, Nanjing, Wuxi, Changzhou, Suzhou, Nantong, Yancheng, Yangzhou, Zhenjiang, Taizhou (in Jiangsu Province), Hangzhou, Ningbo, Jiaxing, Huzhou, Shaoxing, Jinhua, Zhoushan, Taizhou (in Zhejiang Province), Hefei, Wuhu, Maanshan, Tongling, Anqing, Chuzhou, Chizhou and Xuancheng, with an area of 2,117,000 square kilometres (Fig 2).

### Model specification

According to the model of neoclassical economic growth theory, this paper calculates the impact of integrated transport efficiency on urban innovation based on the improved production function under the assumption of constant returns to scale. The simple formula is as follows:

$$\ln Y_{it}=\sum_{j}\gamma X_{j}+C+\varepsilon$$ (1)

where *Y* represents urban innovation and *X* represents the integrated transport efficiency calculated above. *γ* is the coefficient of the corresponding variable, and the formula also contains some controlled variables. *i* represents the city, *t* represents time, *C* is a constant term, and *ε* is the random error term.

Geographical data are easily influenced by spatial factors, and there are certain correlations between research units. Before the spatial econometric model is established, the proposed model must pass the spatial autocorrelation tests, including global and local spatial autocorrelation tests. In order to judge whether the variables are correlated in space, Moran’s I index is used to conduct the global spatial autocorrelation analysis. The calculation formula is as follows:

$$I=\sum_{i=1}^{n}\left(x_{i}-\bar{x}\right)\sum_{j=1}^{n}W_{ij}(x_{j}-\bar{x})/\sum_{i=1}^{n}{\left(x_{i}-\bar{x}\right)}^{2}\sum_{i=1}^{n}\sum_{j=1}^{n}W_{ij}$$ (2)

where *n* represents the number of cities under study and *X*_*i*_ and *x*_*j*_ represent the specific attribute values of a certain city. *W*_*ij*_ is the spatial weight matrix. Three types of spatial weight matrices are adopted in this paper. The adjacency weight matrix (*W*_*r*_) is set according to whether the two cities are adjacent. When two cities are adjacent, the value is equal to 1; otherwise, the value is equal to 0. The time distance weight matrix (*W*_*t*_) refers to the shortest time distance between two cities. It is often used to measure the correlation of cities in transportation. The economic spatial distance weight matrix (*W*_*e*_) uses the geographical distance between the geometric centres of the two cities multiplied by the economic distance, which is the difference between the real GDP per person of the two cities. These matrices have all been standardized. The value of Moran’s I is between -1 and 1. If the value exceeds 0, it represents a positive spatial autocorrelation; conversely, it means the opposite.

The local spatial autocorrelation test can describe the variation characteristics in a certain city. We use the *Getis*−*Ord* $Z(G_{{}_{i}}^{*})$ model to analyse the specific area and its urban agglomeration characteristics.

$$Z(G_{{}_{i}}^{*})=[G_{{}_{i}}^{*}-E(G_{{}_{i}}^{*})]/\sqrt{Var(G_{{}_{i}}^{*})}$$ (3)

where *i* denotes the specific city. If the value of $Z(G_{{}_{i}}^{*})$ exceeds 0 and is significant, the city belongs to a high value spatial agglomeration; otherwise, the city belongs to a low value spatial agglomeration.

Considering the spatial characteristics of integrated transport efficiency, the main spatial econometric models are the spatial error model (SEM), the spatial autoregression model (SAR) and the spatial Durbin model (SDM). Their formulas are as follows, respectively:

Y=ηWY+Xβ+ε (4)

$$Y=\alpha +\beta X+\varepsilon ,\varepsilon =\lambda W_{\varepsilon }+\mu$$ (5)

$$Y_{it}=\eta W_{ij}Y_{jt}+X_{it}\beta +\theta W_{ij}X_{jt}+\mu_{i}+\lambda_{t}+\varepsilon_{it}$$ (6)

where *Y* is the explained variable, *X* is the input variable, *WY* and *WX* represent the spatial dependence, *α* is the constant term, and *ε* is the random error term.

After incorporating the spillover effect of integrated transport efficiency, we take the logarithmic form of each variable and establish the spatial econometric model:

$$\begin{array}{l} lnY_{it}=\beta_{j}\sum_{j}q_{j}X_{j}+\beta_{1}\ln K_{it}+\beta_{2}\ln H_{it}+\beta_{3}\ln SI_{it}+\beta_{4}\ln U_{it}+\beta_{5}\ln O_{it}+\theta_{k}W_{ij}\sum_{k}q_{k}X_{k}\\ +\theta_{1}W_{ij}\ln K_{jt}+\theta_{2}W_{ij}\ln H_{jt}+\theta_{3}W_{ij}\ln SI_{jt}+\theta_{4}W_{ij}\ln U_{jt}+\theta_{5}W_{ij}\ln O_{jt}+\delta W_{ij}Y_{jt}+C+\varepsilon \end{array}$$ (7)

where *δ* is the spillover effect of the integrated transport efficiency of neighbouring cities on local innovation, *β* is the coefficient of the elasticity of variables, *θ* is the coefficient of the spatial matrix, and *ε* is a random error term. If *δ*≠0, *β* and *θ* cannot represent the spatial effect of the explanatory variable.

### Data

#### Selection of variables

Explained variable: Urban innovation (*Y*) represents the annual number of authorized patents from each city in the Yangtze River Delta. There are two types of indicators to measure regional innovation: one is the innovation factor index, which is divided into an input factor and an output factor, and the other is the innovation efficiency index [40]. As the results of knowledge production and technological innovation, patents have become widely used to evaluate urban innovation [41]. Patents include invention patents, utility model patents and design patents, among which invention patents are the most creative and innovative and are the research object of this paper.

Explanatory variable: Integrated transport efficiency (*ITE*) represents the annual efficiency of each city in the Yangtze River Delta, which is calculated in the parts below.

Controlled variables:

Capital stock (*K*) is estimated by the perpetual inventory method. We refer to the formula *K*_*it*_ = *K*_*i*,*t*−1_(1−*λ*_*t*_)+*I*_*it*_/*E*_*it*_, where *K*_*it*_ and *K*_*i*,*t*−1_ represent the capital stock of city *i* in year *t* and year *t*−1, respectively. *E*_*it*_ is the fixed asset investment price index of city *i* in year *t*, and *λ*_*t*_ is the depreciation rate of fixed assets in year *t*. In this paper, a 10.96% depreciation rate is adopted for standardization.

Human capital (*H*) is based on the total number of employed people in each city multiplied by the average number of education years of residents. We refer to Wang to calculate the years of education of residents, and the formula is *E* = 6*S*_1_+10*S*_2_+16*S*_3_. *S*_1_, *S*_2_ and *S*_3_ are the numbers of students in primary school, middle school, colleges and universities per ten thousand people, respectively [42].

The structure of the industry (*SI*) is expressed as the proportion of secondary industry in the GDP of a city.

The degree of urbanization (*U*) is measured by the proportion of the municipal population with respect to the total permanent population of the city.

The degree of opening to the outside (*O*) is the sum of the dependence on foreign capital and foreign trade. Among the components, the dependence on foreign capital is measured by the proportion of actual foreign investment with respect to actual GDP, and the dependence on foreign trade is measured by the proportion of the total foreign import and export trade value with respect to actual GDP. The total value of foreign import and export trade is unified in dollars and converted according to the average exchange rate of the corresponding year.

The paper takes 26 cities in the Yangtze River Delta from 2010 to 2019 as the research units. Relevant city data come from the Urban Statistical Yearbook. Table 1 contains the descriptive statistics for each variable included in this study.

**Table 1:** Descriptive analysis of data.

Variable	*Y*	*ITE*	*K*	*H*	*SI*	*U*	*O*
Mean	8.621	0.915	7.285	5.522	0.513	4.045	0.508
Std. Dev.	1.599	0.170	0.939	0.852	0.072	0.215	0.423
Min	3.178	0.480	3.991	2.415	0.298	3.434	0.065
Max	11.496	1.320	8.818	7.222	0.747	4.495	2.922

### Evaluation of integrated transport efficiency

The efficiency of the integrated transport industry is a comprehensive index to measure the operation of the entire transportation system and the allocation of transportation infrastructure elements. Due to its multidimensional time, space, object and value characteristics, the existing literature focuses on different perspectives and methods [43]. Considering the subjectivity and dispersion of the evaluation results and other factors, many scholars tend to use three models to analyse transportation efficiency: data envelopment analysis (DEA), stochastic frontier analysis (SFA) and neural network analysis (NN).

The DEA model is a quantitative analysis method used to evaluate the relative effectiveness of the same type of comparable units by using a linear programming method based on multiple input indicators and multiple output indicators. This method has been widely used in different industries and departments and shows unique advantages in handling multiple inputs and multiple outputs. Therefore, we apply the DEA model to calculate the efficiency of the urban integrated transport industry. However, if the traditional CCR or BCC model is adopted, the most efficient DMUs will have a value of 1. In order to compare and select the effective units in the optimal frontier, the super-efficiency DEA model proposed by Andersen and Petersen is used to rank the efficient DMUs and retain the inefficient DMUs [44], so that a comparison among DMUs is conducted. The super-efficiency DEA model is as follows:

$$\begin{array}{l} min[\theta -\varepsilon (e_{1}^{T}s^{-}+e_{2}^{T}s^{+})]\\ s.t.\left\{ \begin{array}{l} \sum_{j=1,j\neq 0}^{n}X_{m1}\lambda_{m}+s^{-}=\theta X_{1}^{n};I=1,2,\ldots ,L\\ \sum_{j=1,j\neq 0}^{n}Y_{mk}\lambda_{m}-s^{+}=Y_{{}_{1}}^{m};k=1,2,\ldots ,K\\ \lambda_{m}\geq 0;m=1,2,\ldots ,N-1,N+1,\ldots ,M\\ s^{-}\geq 0;s^{+}\geq 0 \end{array}\right. \end{array}$$ (8)

where *X*_*m*1_ denotes the first input index of the m-th DMU, *Y*_*mk*_ denotes the k-th output index of the m-th DMU, and *θ* denotes the comprehensive efficiency index of the input index and output index. This model calculates the efficiency of the evaluated DMUs through the frontier of other DMUs, and the value of the efficient unit is no longer limited to 1 for comparison purposes. However, for ineffective DMUs, their values will remain unchanged.

The efficiency of the integrated transport industry is often influenced by diverse factors. According to the integrated development plan of higher-quality transportation in the Yangtze River Delta (2021–2035), integrated development includes four aspects, namely, the transportation network, transportation service quality, business mode, and operating mechanism [45]. Among these aspects, the integrated transport network is composed of highways, railways, waterways, aviation and other types of transportation infrastructure. Because transportation network density and accessibility are quantifiable indicators, the mileage of the transportation mode is often used as an input variable, while capital input and energy input are ignored [46].

Therefore, we take fixed investment in the field of transportation infrastructure as the capital input variable, and the perpetual inventory method is adopted to adjust the value. Furthermore, transportation energy consumption is added as the energy input index. Since different cities adopt various measurement standards such as petroleum and natural gas in their energy consumption statistics, in order to achieve a unified comparison, all types of energy resources are converted according to the standard coal conversion coefficient and then standardized.

Passenger and freight volumes, which reflect traffic scale, are mostly used as output variables in previous studies. For a better description of the efficiency of the integrated transport industry, we choose the turnover rate to represent both the quantity and quality performance of transportation. In addition, port container throughput is selected as the output index to measure the development situation of the port. Based on the index system of Li et al. [47] and Abadi et al. [48], we finally establish the following index system (Table 2).

**Table 2:** Index system of integrated transport efficiency.

Classification	Variables	Specific indicators	Unit
Input indicators	Network factor	Expressway percentage of highway	%
		Extended length of railway	km
		Mileage of inland waterway	km
		Number of flights	10000 sorties
		Number of berths	set
	Labor	Number of employees in transportation	10000 persons
	Equipment	Number of operating cars and ships	set
	Capital	Investment in fixed assets in the field of transportation	10000 CNY
	Energy	Energy consumption in transportation	10000 kwh
Output indicators	Traffic	Turnover of the passengers	100 million persons km
		Rotation volume of freight transport	100 million tons km
		Port container throughput	10000 TEU
	Capital	Added value of GDP in transportation	10000 CNY

Considering the availability and timeliness of the data, the paper takes cities as the research unit to measure the integrated transport efficiency of 26 cities in the Yangtze River Delta. Relevant traffic data mainly come from the Urban Traffic Yearbook, the White Paper on Traffic Development, the Statistical Bulletin of the Traffic Industry and the Traffic Operation Analysis Report. In addition, economic data come from the Urban Statistical Yearbook, and energy data come from the China Energy Statistical Yearbook.

Although the integrated transport industry network is a comprehensive system composed of a variety of transportation modes, there are many unreasonable routes in the pipelines, such as many different oil routes, and most data on urban pipeline transportation are lacking. Therefore, we exclude pipelines from transportation modes. However, this does not mean that pipeline transportation does not play an important role in the integrated transportation network, and we expect to consider this important factor in future research.

## Results

### Analysis of integrated transport efficiency

#### Estimation results

**Fig 3:**
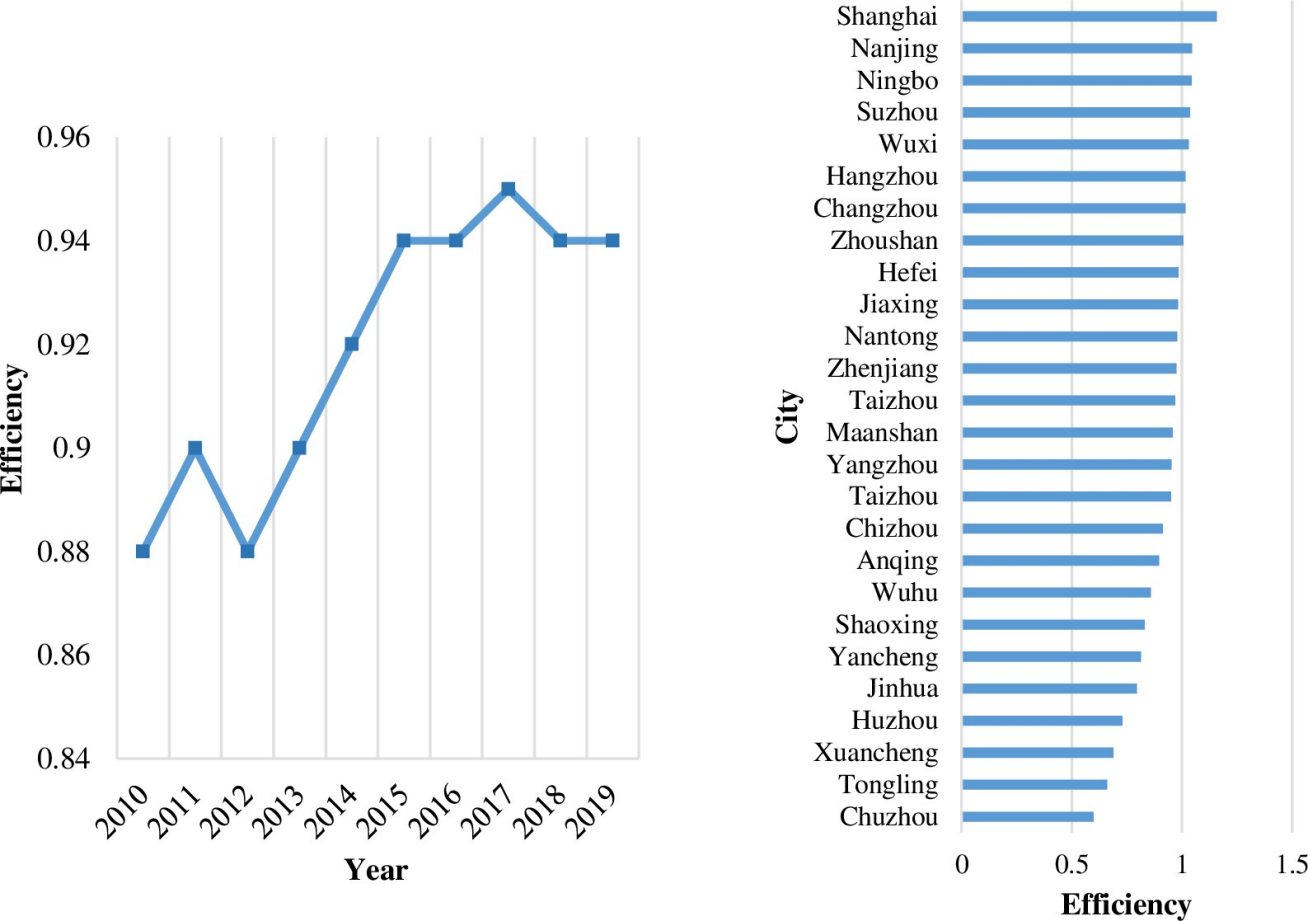
Efficiency measurement results of the integrated transport in the Yangtze River Delta. The data above are calculated by MYDEA 3.0. Taizhou, which is ranked 13, is in Jiangsu province, and Taizhou, which is ranked 16, is in Zhejiang province.

This paper estimates the integrated transport efficiency of 26 cities in the Yangtze River Delta from 2010 to 2019. In order to better compare the efficiency of different cities in the urban agglomeration, their average values in these 10 years are sorted. In addition, the overall efficiency of the urban agglomeration in different years is calculated. The evaluation results are shown in Fig 3.

During the 2010–2019 period, there was an obvious growth trend of integrated transport efficiency, and the average value was approximately 0.92 in the Yangtze River Delta. As central cities in the urban agglomeration, Shanghai, Nanjing and Suzhou maintain a rising trend, and their efficiencies are all above 1.03. The efficiency of some cities surrounding Shanghai, such as Changzhou and Wuxi, shows a trend of first rising, then decreasing and finally rising again. Some cities with high efficiency, which are not close to Shanghai, also benefit greatly from neighbouring central cities. The efficiency of Yancheng, Nantong, Taizhou (in Zhejiang Province) and other cities in coastal areas is flat and slowly improves. With the gradual clarification of the positioning from relevant planning, these types of cities further integrate into the transportation network with Shanghai as the centre, and their integrated transport efficiency will be significantly increased. Yangzhou, Jinhua and other cities located in the geographical centre of the province should have location advantages, but in fact, due to the constraints of the interior economic conditions and the competition from the surrounding cities, their efficiency has been at approximately 0.80 for years.

#### Analysis of the efficiency results

Generally, the top five cities are mainly the leading cities in the Yangtze River Delta, and they have better conditions for economic development. The average value of integrated transport efficiency in Shanghai and other leading cities exceeds 1.15. Leading cities attract increasingly more immigrants due to their strong economic foundations, which results in higher requirements for tourism and circulation. In addition, the increasing traffic demand forces the cities around the leading cities to improve their urban traffic networks as soon as possible. These cities need to expand their production and improve their production efficiency, and an efficient and low-cost integrated transport system will help them enter an era of better development.

Cities with values that exceed 1 comprise nearly 1/3 of the cities studied, and most of them are close to the leading cities in the Yangtze River Delta. Because of the connections of various transportation modes in the Yangtze River Delta, these cities undertake the freight demand brought by the industrial transfer of leading cities and better meet the commuting needs of the reverse flow of the urban population. The connections to large cities not only improve the transport structure of leading cities on the periphery but also promote the efficient development of their own transport networks. Additionally, compared to the development conditions of other cities, such connections improve integrated transport efficiency, which makes the cities in the urban agglomeration more tightly connected.

In addition, most effective cities have the necessary natural conditions. Regarding the natural conditions, coastal cities have better basic conditions than inland cities, such as ports and shipping, which play important roles in the urban integrated transport system. Taking Zhoushan as an example, a reasonable geographical location is conducive to the efficiency improvement and scale expansion of port transportation. In recent years, with the continuous improvement of port foundations, their transport efficiency has reached approximately 1.05 and still has great potential to increase in the future.

In conclusion, it can be seen that most of the cities with a high efficiency keep a leading edge on the economic scale of the cities. The economic foundation is a vital force for promoting the development of urban transportation. The most critical reason for the improvement of the efficiency of the integrated transport industry in the Yangtze River Delta may be that it performs well due to its location advantage in foreign trade. Moreover, integration planning in the Yangtze River Delta brings policy advantages into play, which allow all cities to make good use of their advantages in the existing transport system and connect to the regional transport network in a better way. On this basis, the economic differences between the cities are narrowing, and the density of the transportation network is gradually being balanced.

### Spatial spillover effects results

#### Spatial autocorrelation test results

Through the global autocorrelation test of urban innovation and integrated transport efficiency in the Yangtze River Delta, the results show that the two indicators are both positively correlated under the three types of weight matrices, and the Moran’s I coefficient is the highest in the economic spatial distance weight matrix and the lowest in the time distance weight matrix, which illustrates that the influence of economic factors on the spatial dependence between cities cannot be ignored. Under the economic spatial distance weight matrix (Table 3), the Moran’s I coefficient of the innovation transformation index since 2013 has passed the significance test at the 5% level, with an average value of approximately 0.15, indicating that its spatial agglomeration effect in the Yangtze River Delta is gradually enhanced. The global autocorrelation test result of integrated transport efficiency is lower than that of the urban innovation index; its Moran’s I coefficient has remained above 0.12, and it shows a growth trend. The result still reflects the agglomeration effect of integrated transport efficiency.

**Table 3:** Global spatial autocorrelation test results of urban innovation and integrated transport efficiency under the economic space distance weight matrix in the Yangtze River Delta.

Year	Urban Innovation			Integrated Transport Efficiency		
	Moran’ I	Z(I)	P-value	Moran’ I	Z(I)	P-value
2010	0.060	0.987	0.162	0.034	0.045	0.482
2011	0.064	0.831	0.203	0.071	0.824	0.205
2012	0.100	1.131	0.129	0.077	0.853	0.197
2013	0.188	1.725	0.042**	0.163	1.490	0.068*
2014	0.167	1.659	0.049**	0.006	0.331	0.370
2015	0.188	1.815	0.035**	0.217	1.862	0.031**
2016	0.190	1.788	0.037**	0.122	1.172	0.121
2017	0.205	1.831	0.034**	0.197	1.011	0.056*
2018	0.228	1.984	0.024**	0.286	2.381	0.009***
2019	0.204	1.827	0.034**	0.186	1.638	0.051*

**Note:** The data are calculated by stata 15.0.

***, **, * refer to 1%, 5%, 10% significance levels respectively.

**Fig 4:**
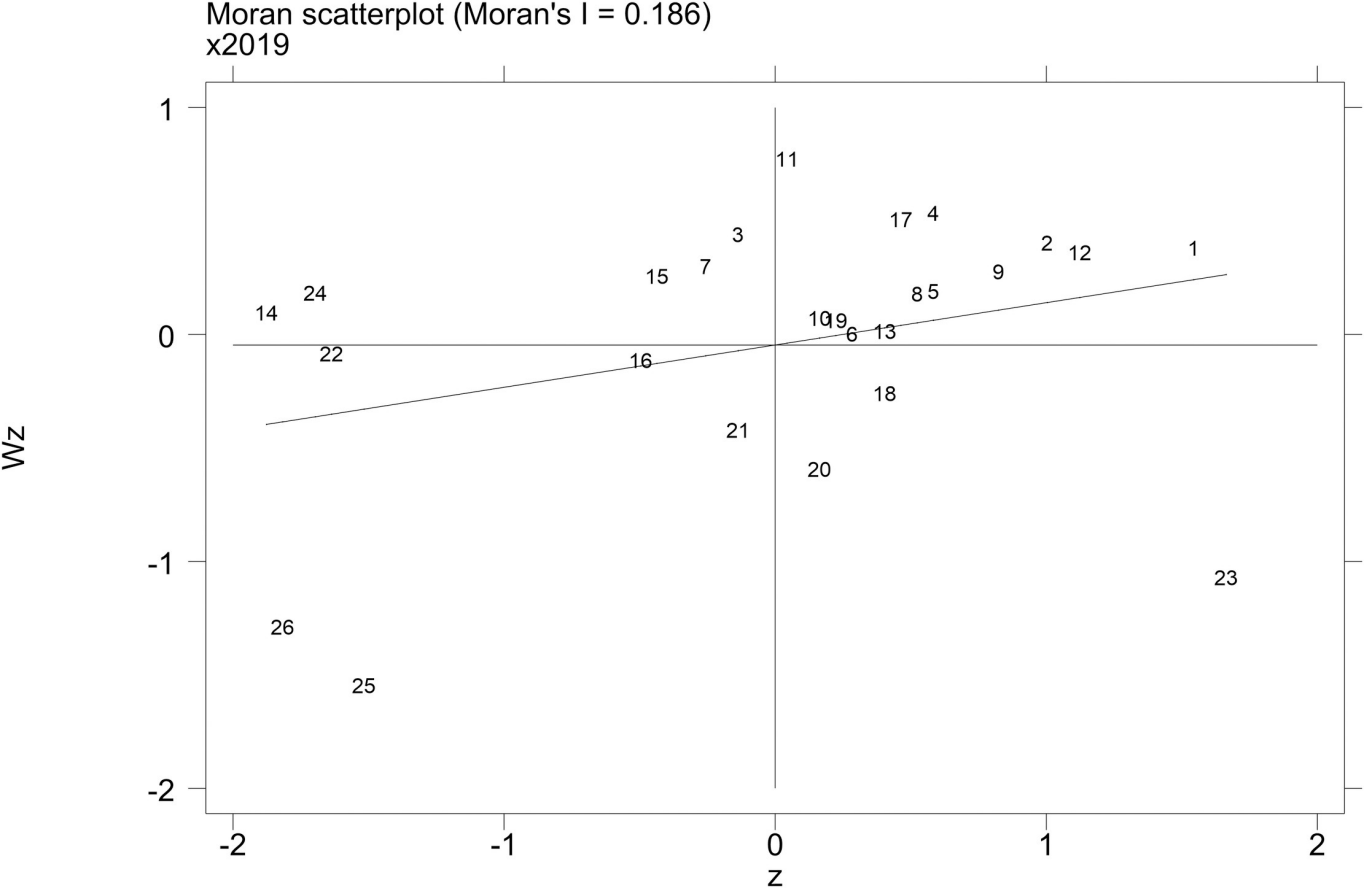
Moran scatterplot diagram of urban innovation and integrated transport efficiency in the Yangtze River Delta in 2019.

**Fig 5:**
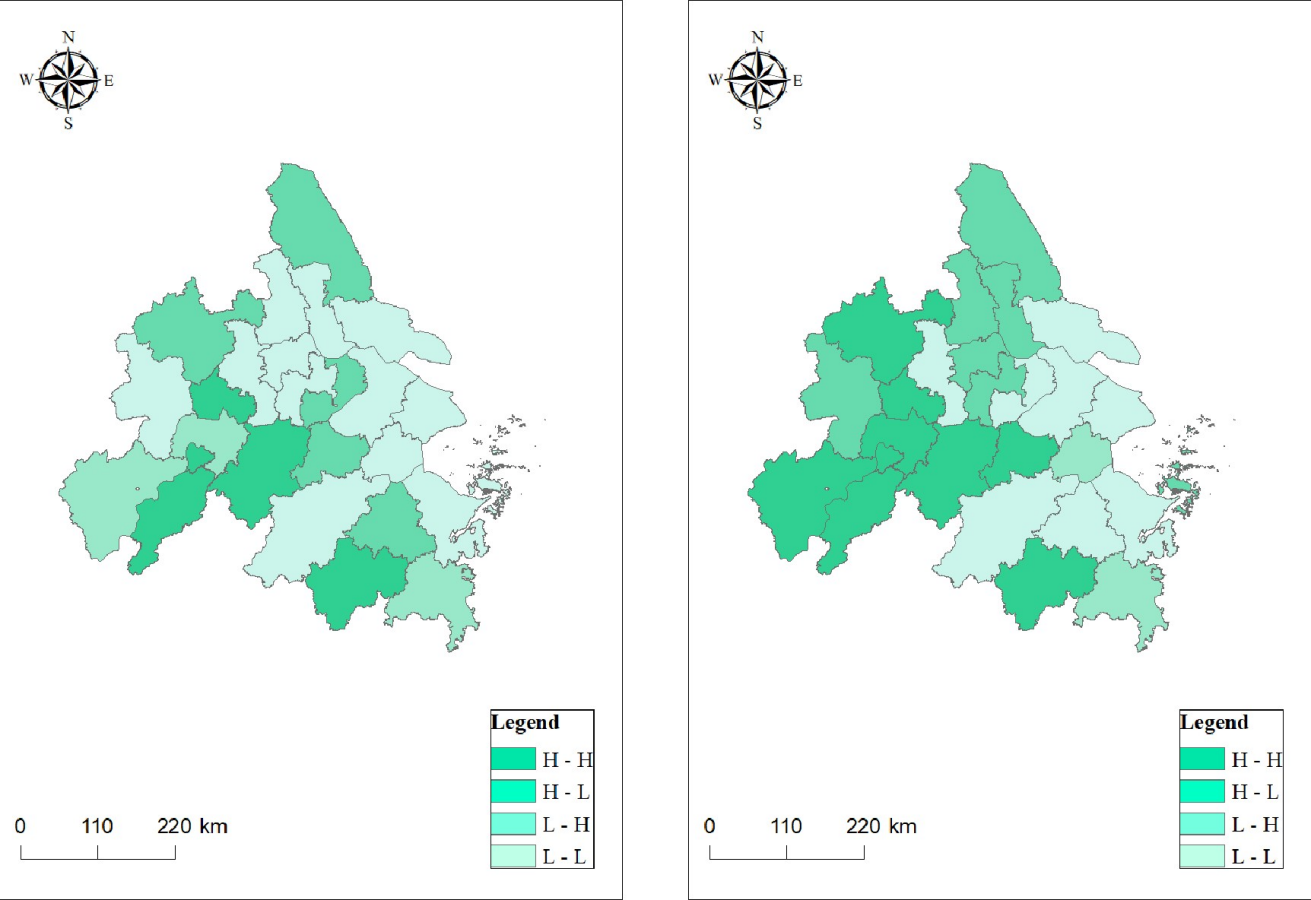
Lisa spatial clustering diagram of urban innovation and integrated transport efficiency in the Yangtze River Delta in 2019. (The data are estimated by the authors themselves and the figure is drawn by Arc GIS).

In order to further test the local spatial autocorrelation of urban innovation and integrated transport efficiency, the paper takes 2019 as an example and explains their spatial distribution statuses using a Moran scatterplot diagram (Fig 4) and a Lisa spatial clustering diagram (Fig 5). The figures show that the distributions of urban innovation and integrated transport efficiency both have significant spatial aggregation effects. In terms of the innovation index, most cities in Anhui Province are located in the L-L agglomeration area, while Shanghai and the southern cities in Jiangsu Province are located in the H-H agglomeration area. In terms of integrated traffic efficiency, 13 cities are located in the H-H agglomeration area, covering most cities in Jiangsu Province. In addition, there are more cities in the L-L and L-H agglomeration areas than in the H-L agglomeration area.

### Comparison of regression results

Without adding the spatial weight matrix, the data are first analysed using an ordinary panel regression (Table 4). The results of the Hausman test show that in the selection of fixed and random effects, the results passed the significance test at the 5% level, which means that the fixed effect model should be used for the estimation. When the spatial panel regression is conducted, we select the fixed effect SDM and compare the R^2^, LR and AIC of the SDM, SEM and SAR. The comparison of the R^2^s show that the degree of fit degree of the urban agglomerations estimated by the SDM is better than that of the ordinary regression, which is consistent with the conclusion of the spatial dependence test. With the introduction of a spatial econometric model, some useful conclusions are obtained in this paper.

**Table 4:** Regression results.

Variables	FE	*W*_*r*_	*W*_*t*_	*W*_*e*_
*ITE*	0.119***	0.262***	0.100	0.286***
*K*	0.676***	0.183**	0.028*	0.187***
*H*	0.643***	0.540***	0.508***	0.764***
*SI*	5.577**	2.324**	4.118***	3.274***
*U*	4.993***	2.215***	1.734***	2.040***
*O*	1.193***	0.363**	0.154*	0.502***
W*ITE	—	0.471*	0.116*	0.292***
W*K	—	0.185*	0.485*	0.601***
W*H	—	-1.231***	-0.573*	-0.184*
W*SI	—	3.989***	4.905***	9.816***
W*U	—	1.557*	5.077**	4.542***
W*O	—	0.827***	0.233	0.954***
*R* ^2^	0.7773	0.8620	0.8658	0.8862
*LR*	—	28.45	25.43	118.88
*AIC*	—	-315.33	-306.00	-247.92

**Note:** The data are calculated by stata 15.0.

***, **, * refer to 1%, 5%, 10% significance levels respectively. FE, *W*_*r*_, *W*_*t*_, and *W*_*e*_ denote the fixed effect of the ordinary panel model, the adjacency weight matrix, the time distance weight matrix and the economic space distance weight matrix, respectively. The estimation results of columns 2 to 4 are the fixed effect results of SDM model. After examination, the SAR model and SEM model cannot be converted to the SDM model.

The existence of spatial correlation factors improves the positive impact of urban integrated transport efficiency on urban innovation. As the results of the main explanatory variable (*ITE)* show, in the general regression model, when the efficiency of the integrated transport industry increases by 1%, the urban innovation transformation will improve by approximately 0.119%. When the spatial factors are considered, the elasticity of the efficiency is almost higher than the result of the general regression, which means that the improvement of efficiency has a significantly positive impact on urban innovation transformation. Therefore, if the spatial correlation factors are ignored, we will underestimate their impact. After comparing the results of the three types of spatial weight matrices, we find that the results of the adjacency weight matrix and the economic spatial distance weight matrix are more significant than those of the time distance weight matrix. Under these weight matrices, when integrated transport efficiency increases by 1%, the urban innovation of the Yangtze River Delta will improve by more than 0.26%, which is mainly due to the significant marginal effect brought by the large-scale effect in the Yangtze River Delta urban agglomeration. The improvement of efficiency reduces the flow costs of elements and products, forms the agglomeration effect of the innovation element flow, and accelerates industrial progress, thus serving regional innovation.

Regarding the spillover effects between the cities, the spillover effects of integrated transport efficiency under the economic spatial distance weight matrix are better than those under the adjacency weight matrix and the time distance weight matrix. The spillover effect of integrated transport efficiency reaches 0.292 under the economic spatial distance weight matrix. That is, the result is larger when economic correlation factors are considered. However, in the time distance matrix, the spillover effect is only 0.116. Therefore, the regional differences in economic development promote the connectivity of the local transport infrastructure, and economic factors play an enhanced role in the process of transforming urban innovation using integrated transport efficiency, but this effect is weakened by geographical and time barriers.

In general, regardless of the type of spatial weight matrix, the spillover effect of the improvement of integrated transport efficiency on the regional innovation of surrounding cities is higher than that of the city itself. Especially in the economic spatial distance weight matrix, both coefficients of urban efficiency pass the significance test at the 1% level. The flow space reflects the mutual connections between cities, which transforms the analysis of the regional spatial structure into a study of the urban network structure, network function and network connections [49]. Therefore, the result of the spillover effect is mainly due to transportation mobility and the transportation network. Improved transport efficiency reduces the transportation costs and enhances local trade mobility. The optimization of the traffic network increases the regional flow efficiency of elements, and the improvement of urban nodes leads to the progress of the innovation network in the Yangtze River Delta.

Based on the reality of urban development in the Yangtze River Delta, the spillover effect brought by the improvement of urban transport efficiency may be caused by the close geographical locations of most cities and more traffic links between cities to some extent. The development of innovation factors in a city, especially the leading city, can be transmitted more quickly through the integrated transport network and then change the agglomeration of factors, resulting in considerable social effects in the region. Considering the future development of the integrated transport system, although the Yangtze River Delta has a complete range of transportation modes, 26 cities are distributed in three provinces and one municipality, and they have obvious differences in transportation planning and transportation operations. Moreover, the planning of regional transportation in the Yangtze River Delta is still in the examination stage, and the construction of transportation infrastructure built in various areas lacks a unified and effective link. These problems may hinder the city from breaking through the provincial boundary to exert a more positive spillover effect.

Among the controlled variables, the main variables are the structure of the industry (*SI*) and the degree of urbanization (*U*), and they both have positive effects on regional innovation in the research period. Each 1% increase in the industrial structure and urbanization rate in the Yangtze River Delta will cause 3.274% and 2.040% growth, respectively, in urban innovation under the economic spatial distance weight matrix. The cities in the Yangtze River Delta basically have manufacturing industry clusters in certain industries. In the urban economic growth process, with the gradual evolution of the industrial structure towards rationalization, the mode and scale of regional manufacturing industry clusters will continue to transform and improve. Through the flow and optimal allocation of resources between cities, production efficiency is improved and production costs are reduced, and thus structural adjustment and regional innovation transformation will be realized. According to urban economics, cities with higher urbanization rates have higher positive externalities, which can generate incentives for manufacturing enterprises to choose locations. An increase in the urbanization rate leads to population concentration; that is, population concentration not only provides sufficient labour for the development of the manufacturing industry but also provides considerable demand for manufacturing products. Therefore, an increase in the urbanization rate has a positive spillover effect in the Yangtze River Delta.

The increase in human capital (*H*) in the Yangtze River Delta has a negative spillover effect on the surrounding cities under the three types of matrices. The increase in the number of labourers and more years of education for residents have alleviated the contradiction between labour resources and demand for efficient economic operations and has a positive effect on local innovation. However, the spillover effects on neighbouring cities are always negative under the three types of weight matrices, which shows that the polarization effect of the surrounding cities exceeds the trickle-down effect. The capital stock (*K*) and the degree of opening to the outside (*O*) both have a positive influence on the innovation development of urban agglomerations. The scale of capital stock determines the realistic foundation and economic strength of urban development and plays an important role in guiding the agglomeration of local innovation elements. In addition, the advantage of government policy provides the Yangtze River Delta favourable conditions for the introduction of foreign capital and the development of trade. The above conclusions show that there is a promising development trend in the Yangtze River Delta in science and technology innovation and cooperation.

## Discussion

For a long time, the scope of Yangtze River Delta urban agglomeration, the location of its boundary, and whether the space will expand outward have been popular topics in political, academic and industrial circles. The number of cities in the Yangtze River Delta is closely related to the overall efficiency of the integrated transport industry. Too large of an agglomeration scale will reduce coordination and communication efficiency between cities, improve the integrated construction cost, and result in higher requirements for the improvement of integrated transport efficiency within the urban agglomeration [50].

In the relevant government planning, the scope of the Yangtze River Delta has been changing over different stages of development (Table 5). In 1982, the State Council of China approved the establishment of the Shanghai economic district, which is divided into 10 cities. In 1997, the forum for the coordination of the urban economy of the Yangtze River Delta, composed of 15 cities, was established. In 2010, the regional plan for the Yangtze River Delta, covering 25 cities in Shanghai, Jiangsu Province and Zhejiang Province, was released [51]. In 2016, the National Development and Reform Commission issued the development planning of Yangtze River Delta urban agglomeration with a total of 26 cities [39]. In December 2019, the State Council of China issued the outline of the integrated regional development of the Yangtze River Delta, which covers all cities in Shanghai, Jiangsu Province, Zhejiang Province and Anhui Province, including 27 central cities [52].

**Table 5:** Planning documents in the Yangtze River Delta.

Reference standard	Year	City included	Details			
			SH	JS	ZJ	AH
Shanghai economic district	1982	10	1	4	5	
The forum for the coordination of urban economy of the Yangtze River Delta	1997	15	1	8	6	
The regional planning for the Yangtze River Delta	2010	25	1	13	11	
The development planning of urban agglomeration in the Yangtze River Delta	2016	26	1	9	8	8
The outline of the integrated regional development of the Yangtze River Delta	2019	41	1	13	11	16

**Note:** SH, JS, ZJ, AH represent Shanghai, Jiangsu Province, Zhejiang Province and Anhui Province respectively.

The results of integrated transport efficiency in the Yangtze River Delta are different due to the different planning documents. It is worth mentioning that if we only select the 10 cities of the Shanghai economic district as the research objects, the average efficiency is approximately 1.06 and higher than the estimation result in this paper. The 10 cities most affected by the radiation of Shanghai are also covered by the early Shanghai metropolitan area. If we calculate the transport efficiency of the 41 cities classified in 2019, the result will drop to 0.90. This result may be because Anhui Province, which is located inland, is restricted by economic conditions, a limited radiation reception and other adverse factors. With the development of regional integration, Anhui Province still has considerable potential to improve the efficiency of the integrated transport system.

Considering that the capital cities of the Yangtze River Delta are the economic, political, cultural and service centres of each province, these cities have become the leaders of regional innovation and economic activities over a long period of time by virtue of their superior policy advantages and other factors. This paper attempts to remove the samples from these four capital cities of Shanghai, Nanjing, Hangzhou and Hefei and applies the same model to estimate the spillover effect of efficiency under the economic spatial distance weight matrix. The coefficients of the main explanatory variable and its spillover effect are both lower than those of the original sample, but the results all pass the significance test at the 5% level. The results show that the improvement of integrated transport efficiency in most cities in the Yangtze River Delta has a significant effect on the innovation transformation of local and surrounding areas, but the role of regional central cities is more important and indispensable.

In conclusion, the city scale is not the necessary condition for the integrated transport efficiency of the Yangtze River Delta, and the construction of the integrated transport system in the leading cities and regional central cities ensures the development of regional innovation. As Anhui Province has been listed in Yangtze River Delta planning in recent years, its transportation has been gradually integrated into the construction of a transportation network, and the transportation system in the western region of the Yangtze River Delta will be gradually improved. However, with the gradual expansion of the radiation scope in the Yangtze River Delta, the improvement of transport efficiency and the balance of development are facing more severe challenges.

## Conclusions and policy recommendations

Based on the theory of flow space, our goal was to explore the spillover effect of integrated transport efficiency on regional innovation in the Yangtze River Delta. To start, the efficiency of the integrated transport industry was calculated by the super-efficiency DEA model, and the results showed that the average efficiency has reached 0.92. Most of the cities with a high efficiency are the central cities within the urban agglomeration, generally have complete transportation modes, and can be provided with an economic foundation or good natural conditions. The improvement of regional transport efficiency requires the cooperation of various types of transportation modes, and any transportation mode cannot be separated from the network system.

After establishing the specific spatial econometric model, there is clear evidence that an increase in integrated transport efficiency has a positive spillover effect on urban innovation in the Yangtze River Delta, and thus the spatial correlation factors cannot be ignored in the discussion. It is worth mentioning that the coefficient of the spillover effect of efficiency on the regional innovation of surrounding cities is higher than that of the city itself, especially in the economic spatial distance weight matrix. Other controlled variables can also improve the transformation of innovation value to some extent. As one of the most dynamic regions of economic development in China, the Yangtze River Delta has great potential to improve the efficiency of the integrated transport industry to achieve high-quality innovation development. In view of current transportation progress in the Yangtze River Delta, this paper provides the following policy suggestions.

First, it is necessary to promote the comprehensive development of the integrated transport industry by incorporating and combining various transport modes. A large number of cities with low efficiency perform well in a certain transportation mode, but their dimensional transportation network has not yet been formed, which hinders the improvement of integrated efficiency. The Yangtze River Delta has a relatively complete network of highways, and high-speed trains connect the cities in a faster way. In addition, the advantages of the shipping industry and port infrastructure bring opportunities for economic growth, and the distribution of international and domestic airports forms a huge aviation network. In the flow space, as an inseparable node, a single city cannot form its own independent system, and all types of transportation modes require the cooperation of the integrated transport network.

Second, it is of great importance to break the administrative barriers near the provincial boundaries and strengthen overall regional planning. The Yangtze River Delta includes three provinces and one municipality. If there is no consensus on unified planning, the boundary will prevent the efficiency of the integrated transport industry from increasing, and then the spillover effect between cities will be restricted by urban administrative development. In the future, the focus of integrated transport efficiency should be on connecting the transportation between provinces. Anhui Province, as the link between central and western China, should be given full play to develop its linking role. Under policy guidance, the transportation system has become relatively mature, and the radiation effect of the central city is becoming increasingly significant.

Finally, the transportation connection between the leading cities and their surrounding cities should be coordinated to maximize efficiency. The empirical results of this study show that improving transport efficiency will bring a more significant positive spillover effect to the surrounding areas. The central cities should improve efficiency, reduce consumption and deepen integration, which will lead the integrated transport system to create a new era of innovation. The surrounding cities need to use the spillover effect of central cities in the region and integrate resources in various places and potential zones. Furthermore, they should refine the division of labour to promote the complementarity of their advantages, implement a reasonable transfer of regional innovation centres and form a new pattern of balanced development within urban agglomerations. Thus, cities in the Yangtze River Delta will be connected to an integrated network that consists of various transportation modes and actively play a role in the agglomeration effect and spillover effect, which will become the driving force of innovation development in China.

## Supporting information

S1 File Map data. (RAR)

S2 File Original data. (ZIP)
